# Empagliflozin-Metformin Combination Has Antioxidative and Anti-Inflammatory Properties that Correlate with Vascular Protection in Adults with Type 1 Diabetes

**DOI:** 10.1155/2022/6796470

**Published:** 2022-05-17

**Authors:** Miodrag Janić, Matej Cankar, Jan Šmid, Alenka France Štiglic, Aleš Jerin, Mišo Šabovič, Andrej Janež, Mojca Lunder

**Affiliations:** ^1^Clinical Department of Endocrinology, Diabetes and Metabolic Diseases, University Medical Centre Ljubljana, Zaloška 7; SI-1000 Ljubljana, Slovenia; ^2^Faculty of Medicine, University of Ljubljana, Vrazov trg 2, SI-1000 Ljubljana, Slovenia; ^3^Institute of Clinical Chemistry and Biochemistry, University Medical Centre Ljubljana, Njegoševa 4, SI-1525 Ljubljana, Slovenia; ^4^Faculty of Pharmacy, University of Ljubljana, Aškerčeva 7, SI-1000 Ljubljana, Slovenia; ^5^Clinical Department of Vascular Diseases, University Medical Centre Ljubljana, Zaloška 7; SI-1000 Ljubljana, Slovenia

## Abstract

**Methods:**

40 individuals with type 1 diabetes (average age of 44.7 ± 2.5 years) were randomized into four groups: (1) control (placebo), (2) empagliflozin 25 mg daily, (3) metformin 2000 mg daily, and (4) empagliflozin-metformin combination (25 mg and 2000 mg daily, respectively). At inclusion and after 12 weeks of treatment, the blood samples were collected, and the oxidative stress (total antioxidative status (TAS), superoxide dismutase (SOD), glutathione peroxidase (GPx), uric acid, advanced oxidation protein products (AOPP), advanced glycosylation end products ((AGE) and isoprostane), and inflammation (C-reactive protein (CRP) and interleukin-6 (IL-6)) parameters were determined.

**Results:**

The empagliflozin-metformin combination increased levels of the antioxidants (TAS, SOD, and GPx up to 1.1-fold; *P* < 0.01), decreased the levels of prooxidants (AOPP and isoprostanes up to 1.2-fold, *P* < 0.01; AGE up to 1.5-fold, *P* < 0.01), and decreased inflammatory parameters (up to 1.5-fold, CRP *P* < 0.01; IL-6 *P* < 0.001). Antioxidative action was associated with the improvement in arterial function (obtained in the previous study) in the empagliflozin-metformin combination group.

**Conclusion:**

Empagliflozin-metformin combination has strong antioxidative and anti-inflammatory capacity, in adults with type 1 diabetes that is greater than that for the individual drugs. Its antioxidative activity at least partially explains the improvement in arterial function. Therefore, it appears that the combination provides the most powerful vascular protection.

## 1. Introduction

Chronic hyperglycaemia and glycaemic variability due to long-standing diabetes increase the risk of chronic complications. A major vascular complication in diabetes is accelerated atherosclerosis, preceded by impaired endothelial function and increased arterial stiffness as preclinical manifestations [1]. Cardiovascular diseases are, globally, the leading cause of morbidity and mortality, and they significantly diminish the quality of life and lead to premature mortality in diabetes [2–4]. Therefore, interventions that could halt or slow down these processes even before atherosclerotic manifestations are of utmost importance and highly desired, particularly in this patient population. Previously, for the first time, we found that the empagliflozin-metformin combination had the most powerful effect in improving arterial function in type 1 diabetes. In detail, the empagliflozin-metformin combination significantly improved endothelial function (measured by flow-mediated dilation (FMD) and reactive hyperaemia index (RHI)) and decreased arterial stiffness (measured as pulse wave velocity (PWV) and local carotid artery stiffness (*β*-stiffness)). The mechanism behind these promising findings have not yet been completely understood [5].

Hyperglycaemia accelerates atherosclerosis through several mechanisms, the most prominent being the increased oxidative stress and enhanced activation of inflammatory pathways [1]. In atherosclerosis and other chronic vascular conditions, the level of reactive oxygen species (ROS) increases, causing damage to proteins, lipids, and DNA fragmentation. Further cell and organ damage are caused by inflammation [6]. Both, sodium glucose transport protein 2 (SGLT-2) inhibitors and metformin possess several beneficial pleiotropic effects. SGLT-2 inhibitors, besides their action on glycaemic control, act favourably on the vasculature and have important antioxidative and anti-inflammatory actions [4, 7–14]. In type 2 diabetes patients with established ischaemic heart disease and post coronary artery bypass grafting (CABG), SGLT-2 inhibitors ameliorate clinical outcomes and reduce inflammatory burden [15]. Even more, treatment with SGLT-2 inhibitors leads to a more stable carotid atherosclerotic plaque phenotype in type 2 diabetes patients [16]. Metformin also improves endothelial function and decreases urinary isoprostane, a marker for oxidative stress, in individuals with type 1 diabetes [17]. It also reduces ROS production and upregulates endothelial nitric oxide synthase (eNOS), improves mitochondrial function, deactivates polyol pathway, reduces the formation of advanced glycation end products (AGEs), deactivates protein kinase C (PKC), reduces endothelial inflammation, apoptosis, and senescence, and regulates microRNAs (miRNAs) [18, 19]. Furthermore, metformin reduced inflammatory parameters, expression of SGLT-2, and leptin levels from pericoronary fat through the improvement of sirtuin 6 (SIRT6), in people with prediabetes undergoing CABG due to acute myocardial infarction [20].

Based on previous data, the beneficial anti-inflammatory and antioxidative pleiotropic effects of empagliflozin and metformin [4, 7–13, 17, 18] are evident, but their combined action on inflammation and oxidative stress have not been studied yet. Furthermore, the associations between antioxidative and anti-inflammatory activity on one hand and the improvement of arterial function on the other hand are still not fully understood. Therefore, the first aim of the present study was to explore the antioxidative and anti-inflammatory capacity of empagliflozin-metformin combination in comparison to the individual drugs or placebo. The second aim was to analyse the associations between these parameters and improvement in the arterial function. Oral antidiabetic treatment-naïve adults with type 1 diabetes were chosen as a characteristic patient population model, enabling us to study the pure effects of the empagliflozin-metformin combination.

## 2. Materials and Methods

### 2.1. Study Design

This study was designed as a double-blind, randomized, interventional study. Forty individuals with type 1 diabetes from the outpatient clinic at University Medical Centre Ljubljana were recruited. They were equally randomized into four groups, as follows: (1) control (placebo), (2) empagliflozin (25 mg daily), (3) metformin (2000 mg daily), and (4) empagliflozin-metformin (empagliflozin 25 mg daily and metformin 2000 mg daily). The treatment period lasted 12 weeks. Participants took study medications in addition to their standard insulin (multiple daily insulin injections or insulin pump) therapy. All individuals participated voluntarily; signed informed consent was obtained from all participants. The study was performed following the ethical standards of the Declaration of Helsinki and was approved by the National Medical Ethics Committee of Slovenia. It is registered at http://clinicaltrials.gov (NCT03639545).

### 2.2. Study Population

Inclusion criteria consisted of confirmed type 1 diabetes diagnosis with glycated haemoglobin (HbA1c) above 7.0%, age 30-65 years, and body mass index (BMI) over 25 kg/m^2^. Exclusion criteria were HbA1c below 7.0%, age below 30 years or above 65 years, and body mass index (BMI) below 25 kg/m^2^. Additionally, patients with important comorbidities such as advanced heart failure (left ventricular ejection fraction below 40%, NYHA class II-III, NT-proBNP values above 500 pg/mL), kidney (estimated glomerular filtration rate below 60 mL/min/1.73 m^2^) or liver failure (increased transaminases three times above normal value), clinically important benign prostatic hyperplasia, prostatic carcinoma, or history of frequent urinary tract infections were also not included.

### 2.3. Study Protocol

The participants were assessed at inclusion and after 12 weeks of treatment; no further follow-up was performed. At inclusion, a complete medical history and full medical examination were performed. At these two time points, venous blood samples and second morning urine samples were also collected for additional analyses. In addition, ultrasonographic and morphological arterial function assessments were performed at both times. A single operator who was blinded for the study protocol and treatment performed the measurements. The methods were observer-independent; the results were generated automatically. Endothelial function was assessed ultrasonographically by performing the brachial artery flow-mediated dilation (FMD) and tonometrically measuring the reactive hyperaemia index (RHI). Arterial stiffness was also determined ultrasonographically and tonometrically through pulse wave velocity (PWV) and common carotid artery stiffness (*β*-stiffness) measurements, as described previously [5]. Blood pressure measurements were also performed, using an automated sphygmomanometer. All the anthropometric measurements were performed by one operator; participants undressed down to their underwear. All measurements were taken twice, and in case of significant variation between the two readings, a third measurement was performed.

### 2.4. Study Endpoints

The first endpoint of the present study was to determine the possible antioxidative and anti-inflammatory capacity of the empagliflozin-metformin combination in comparison to the individual drugs or placebo in type 1 diabetes patients. The second endpoint covered associations between antioxidative and anti-inflammatory parameters in the empagliflozin-metformin combination group and improvement in arterial function. The latter were the main objectives of our previous study, where a detailed analysis of arterial function was performed [5].

### 2.5. Analysis of Blood Samples

This study was designed as a continued analysis of the stored blood samples that were collected at the inclusion of the study and after 12 weeks of treatment. Blood glucose and HbA1c were determined using the VITRO 5.1FS Chemistry System (Ortho Clinical Diagnostics, Raritan, New Jersey).

#### 2.5.1. Determination of Serum Antioxidants

Total antioxidant status (TAS) in serum was measured by suppression of 2,2′-azino-di-(3-ethylbenzothiazoline-6-sulphonate) radical cation (ABTS) production in the presence of hydrogen peroxide with antioxidants from the sample. The absorbance was measured at 600 nm (Olympus AU400, Mishima Olympus co., LTD, Japan; reagent: Total antioxidant status (TAS), Randox Laboratories Limited, United Kingdom).

The glutathione peroxidase (GPx) activity in erythrocytes was measured through a coupled reaction with glutathione reductase (GR). In the assay, cumene hydroperoxide was reduced and glutathione (GSH) oxidized to glutathione disulfide (GSSG) by GPx. Glutathione reductase then reduced the generated GSSG to GSH with the consumption of nicotinamide adenine dinucleotide phosphate (NADPH). The decrease in NADPH is proportional to GPx activity and was measured at 340 nm (Olympus AU400 Mishima Olympus co., LTD, Japan; reagent: RANSEL, Randox Laboratories Limited, United Kingdom).

For the superoxide dismutase (SOD) activity in erythrocytes, a colorimetric method was used. The inhibitory SOD activity in the formation of reduced red formazan dye with superoxide radical and xanthine oxidase from tetrazolium salt was measured at 520 nm (Olympus AU400 Mishima Olympus co., LTD, Japan; reagent: RANSOD, Randox Laboratories Limited, United Kingdom).

Uric acid was determined through an enzymatic reaction with uricase and Trinder-like endpoint product detection. The absorbance of the complex was measured at 545/694 nm.

#### 2.5.2. Determination of the Serum Prooxidants and Proinflammatory Parameters

The concentrations of AGEs, advanced oxidation protein products (AOPP), and interleukin-6 (IL-6) were measured in serum samples. The aliquots for each analyte were analysed in one batch. The AOPP was measured using competitive ELISA (Cloud-Clone Corp., Katy, TX, USA); the limit of detection was 26 *μ*g/L and CV within the series was under 10%. Serum aliquots used for the measuring of AOPP were diluted 200-fold before analysis. Interleukin-6 was measured with high sensitivity sandwich ELISA (BioVendor, Brno, Czech Republic) with a detection limit of 0.8 ng/L and CV within series <5%.

The concentrations of 8-hydroxydeoxyguanosine (8-OHdG), plasminogen activator inhibitor type 1 (PAI-1), AGEs, (AOPP), and IL-6 were measured in serum samples. The aliquots for each analyte were analysed in one batch. Competitive ELISA immunoassay with a detection limit of 2 *μ*g/L and CV within series <6% (CusaBio Biotech Ltd, Houston, TX, USA) was used for the measurement of 8-OHdG. The concentrations of PAI-1 and AGEs were measured with sandwich-type ELISA (CusaBio Biotech Ltd, Houston, TX, USA) with a detection limit of 0.08 *μ*g/L and 0.2 mg/L, respectively; CVs within series were <8% for both assays. Advanced oxidation protein product was measured using competitive ELISA (Cloud-Clone Corp., Katy, TX, USA); the limit of detection was 26 *μ*g/L and CV within series was under 10%. Serum aliquots used for the measurement of AOPP were diluted 200-fold before analysis. The measurement of IL-6 was performed using high sensitivity sandwich ELISA (BioVendor, Brno, Czech Republic) with a detection limit of 0.8 ng/L and CV within series <5%.

### 2.6. Data Collection

Participants were identified by study ID; all blood and urine samples were also labelled by a study ID; the participants remained anonymous throughout the whole course of the study. Study data (medical history and full medical examination) were collected through paper case report forms that were stored securely. The data was also entered into the electronic database on an on-going basis. The results of arterial function measurements were backed up from the measurement devices. Data from laboratory analyses were provided and stored in paper form; they were additionally transferred to the electronic database system of the study. The electronic database was stored in secured hard drives with backup on password-protected computers that were only accessible to the research team.

### 2.7. Statistical Analysis

The values are expressed as mean ± SEM. The statistical analysis was performed by using a one-way analysis of variance (ANOVA). Where significant interactions were found, the Bonferroni posttest was performed. A *P* < 0.05 was considered statistically significant. The possible associations between parameters of arterial function with the parameters of oxidative stress and inflammation were calculated using Pearson's correlation coefficients. The GraphPad Prism 5.0 software was used for statistical analysis performance.

## 3. Results

### 3.1. Participants' Baseline Characteristics

All male participants were aged 44.7 ± 2.5 years with an average duration of type 1 diabetes of 22.6 ± 3.9 years and an average HbA1c of 7.8 ± 0.3%. Their average baseline systolic and diastolic blood pressure values were 131.4 ± 4.1 and 80.1 ± 2.9 mmHg, respectively. In each study group, 20% were smokers and 10% of the patients had suffered from a previous cardiovascular event. Additionally, 41% of the study participants had diabetic retinopathy, 12.5% had diabetic neuropathy, and 22% had diabetic kidney disease. Importantly, the age of the participants, duration of diabetes, glycaemic control, smoking status, and the occurrence of microvascular chronic complications or previous cardiovascular events did not differ between study groups. Baseline characteristics of the enrolled participants are summarized in Table 1.

### 3.2. Serum Antioxidant Levels

**Figure 1:**
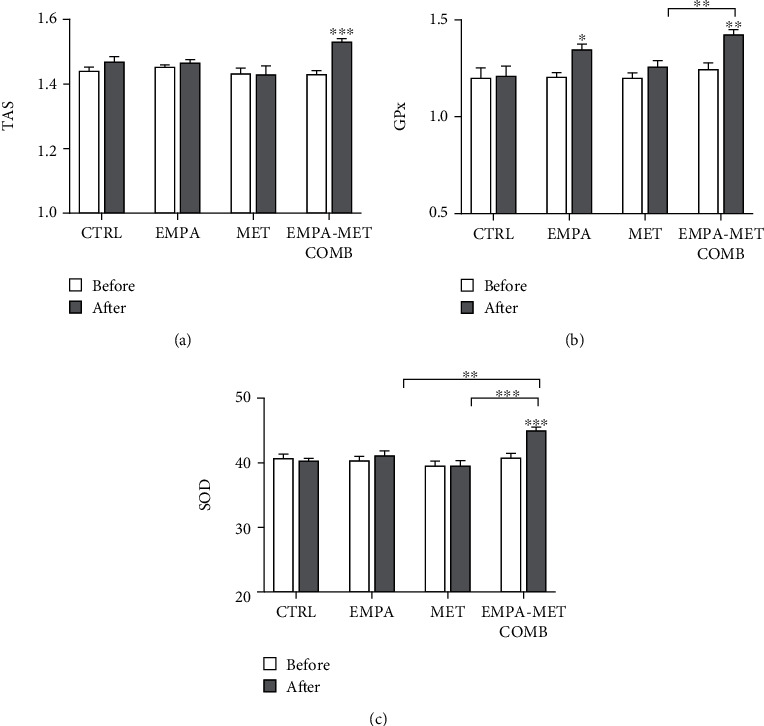
Levels of serum antioxidants in individuals with type 1 diabetes, before and after treatment with empagliflozin 25 mg daily (EMPA), metformin 2000 mg daily (MET) or empagliflozin-metformin combination (EMPA-MET COMB), or in control (CTRL): (a) total antioxidative status (TAS), (b) glutathione peroxidase (GPx), and (c) superoxide dismutase (SOD). ∗ represents *P* < 0.05; ∗∗ represents *P* < 0.01, and ∗∗∗ represents *P* < 0.001.

The empagliflozin-metformin combination significantly increased TAS up to 1.1-fold compared to the control group (*P* < 0.01). The increase of TAS in the combination group was also significant compared to both individual drugs (*P* < 0.05 for both). GPx activity increased in individuals treated with the empagliflozin-metformin combination and in the group treated with empagliflozin only (both up to 1.1-fold; *P* < 0.01) compared to controls. The increase in GPx activity in the combination group was also significant compared to the metformin group (*P* < 0.01). Only the empagliflozin-metformin combination significantly increased the levels of SOD up to 1.1-fold, compared to the control and the individual drugs (all *P* < 0.01). No significant differences in serum antioxidant levels were observed in the control group throughout the study (Figure 1).

### 3.3. Serum Prooxidant and Proinflammatory Parameter Levels

**Figure 2:**
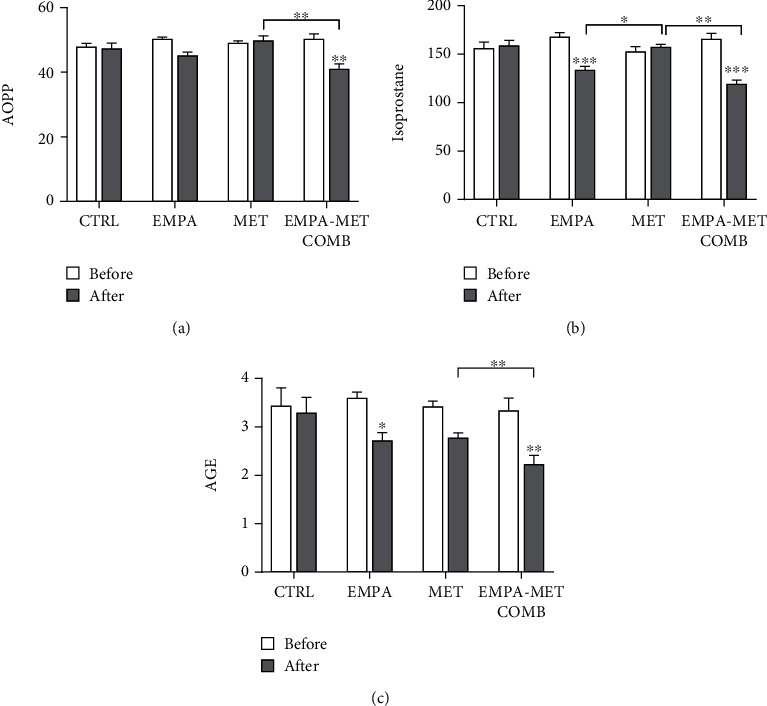
Levels of serum prooxidants in individuals with type 1 diabetes, before and after treatment with empagliflozin 25 mg daily (EMPA), metformin 2000 mg daily (MET), empagliflozin-metformin combination (EMPA-MET COMB), or in control (CTRL) groups: (a) advanced oxidation protein products (AOPP), (b) isoprostane, and (c) advanced glycation end products (AGE). ∗ represents *P* < 0.05; ∗∗ represents *P* < 0.01, and ∗∗∗ represents *P* < 0.001.

The empagliflozin-metformin combination significantly decreased the formation of AOPP by 1.2-fold compared to the control or only metformin (both *P* < 0.01). The levels of isoprostanes and AGEs significantly decreased up to 1.2–fold and 1.5-fold, respectively, in the empagliflozin-metformin combination group and empagliflozin alone compared to only metformin or control (all *P* < 0.01). No significant differences in the prooxidant levels were observed with the controls (Figure 2).

**Figure 3:**
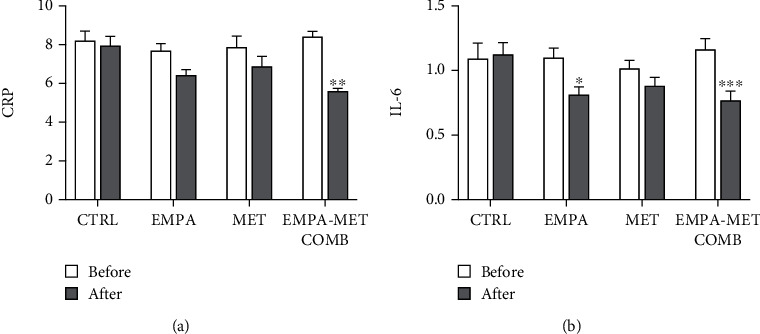
Levels of proinflammatory parameters in individuals with type 1 diabetes, before and after treatment with empagliflozin 25 mg daily (EMPA), metformin 2000 mg daily (MET), empagliflozin-metformin combination (EMPA-MET COMB), or in control (CTRL) groups: (a) C-reactive protein (CRP) and (b) interleukin-6 (IL-6). ∗ represents *P* < 0.05; ∗∗ represents *P* < 0.01, and ∗∗∗ represents *P* < 0.001.

Empagliflozin-metformin combination decreased CRP values up to 1.5-fold compared to the control group (*P* < 0.01). The level of IL-6 significantly decreased up to 1.5-fold (*P* < 0.001) in the empagliflozin-metformin combination group and up to 1.3-fold (*P* < 0.05) in the group treated with empagliflozin only, compared to the controls. No significant differences in the proinflammatory parameter levels were observed in the control group (Figure 3).

### 3.4. Associations between Prooxidative/Proinflammatory and Arterial Wall Parameters

The associations between prooxidative and proinflammatory parameters were studied only in the group treated with an empagliflozin-metformin combination. Brachial artery flow-mediated dilation (FMD) positively correlated with TAS (*R* = 0.38, *P* = 0.009) and negatively with AOPP (*R* = −0.50, *P* = 0.001) and isoprostane (*R* = 0.52, *P* = 0.001). Arterial stiffness parameters also correlated with prooxidative parameters. Pulse wave velocity (PWV) and carotid artery *β*-stiffness are also positively correlated with AGE (*R* = 0.47, *P* = 0.001 and *R* = 0.32, *P* = 0.03, respectively) and isoprostane (*R* = 0.31, *P* = 0.001 and *R* = 0.44, *P* = 0.04, respectively). Carotid artery *β*-stiffness was also positively associated with AGE (*R* = 0.32, *P* = 0.034).

## 4. Discussion

We focused our research on possible antioxidative and anti-inflammatory pleiotropic effects of the empagliflozin-metformin combination in comparison to the individual drugs or control, in adults with type 1 diabetes. This patient group was recruited as a model of long-standing diabetes (of any aetiology) leading to uniform vascular impairment. The participants were oral antidiabetic drug-naïve, thus enabling us to study the effect of the pure drug in a homogenous group. That being said, we believe that the results obtained can be extrapolated to individuals with type 2 diabetes, as vascular impairment mechanisms in these two populations have common denominators. Treatment with empagliflozin-metformin combination increased serum antioxidant levels (TAS, SOD, and GPx) and decreased serum prooxidant levels (AOPP, isoprostanes, and AGE). Furthermore, inflammatory parameters (CRP and IL-6) also decreased after 12 weeks of treatment. The empagliflozin-metformin combination provided the most powerful antioxidative and anti-inflammatory protection, in comparison to the individual drugs. Additionally, the beneficial antioxidative effect of this combination was significantly associated with an improvement in arterial function. Taking all the results into account, we can speculate that the antioxidative and anti-inflammatory action of empagliflozin-metformin combination represents one of the major mechanisms in the improvement of arterial function.

The present study represents a further analysis of the results obtained in our previous study which showed the beneficial effects of the empagliflozin-metformin combination on arterial function in adults with type 1 diabetes [5]. Previously, we had found that after 12 weeks of treatment, the empagliflozin-metformin combination significantly improved endothelial function, defined as increased brachial artery FMD and RHI. The empagliflozin-metformin combination was also superior to metformin in improving arterial stiffness, i.e., reducing PWV and *β*-stiffness [5]. However, the mechanisms behind the observed beneficial protective vascular actions were not studied at that time; the results from the present study represent a logical step forward in the exploration of this important cardiovascular benefit.

According to preclinical studies, improvement of vascular function with SGLT-2 inhibitors can be mediated through reduction of inflammation, vascular oxidative stress, and an increase in membrane hyperpolarization [4, 7–9]. As these pathophysiological processes play an important role in atherosclerosis, we sought to determine if the effect on them could be linked to the improvement of vascular function with the empagliflozin-metformin combination, as previously established. Bearing in mind that both drugs could possess antioxidative as well as anti-inflammatory activity, we presumed that combining the two would yield the greatest benefit. Thus far, the effects of empagliflozin-metformin combination on inflammation as well as oxidative stress have not been studied. Furthermore, the association between the observed antioxidative and anti-inflammatory effects of SGLT-2 inhibitors or metformin and improvement of vascular function parameters have not yet been fully elucidated.

Several previous studies have proved the antioxidative and anti-inflammatory action of SGLT-2 inhibitors in preclinical and clinical studies, but the effect of combining it with metformin has not been studied. Canet et al. found that empagliflozin 10 mg daily for 12 weeks showed antioxidative and anti-inflammatory properties in individuals with type 2 diabetes, and the results were in accordance with a decrease in adhesion molecules and a reduction in leukocyte-endothelium molecules interaction. They concluded that these actions might underlie the beneficial cardiovascular effects [12]. Similar findings were found in another study with empagliflozin 25 mg daily for 24 weeks where the inflammatory markers were studied [21]. All these findings are also in line with the study by Iannantuoni et al., where empagliflozin 10 mg daily for 12 and 24 weeks was studied according to standard care [13]. It should be kept in mind that these studies were performed on individuals with type 2 diabetes and metformin was not a part of the treatment plan [12, 13, 21]. Furthermore, we should also appreciate that the distribution of the SGLT-2s is widespread throughout the body. They can be found in the endothelium [14], atherosclerotic plaques [16], pericoronary fat [20], and cardiac cells [22]. It has been shown that inhibiting or diminishing their expression with either SGLT-2 inhibitors or metformin could lead to favourable clinical outcomes through an improvement in endothelial function, inflammatory, and oxidative stress reduction as well as inhibition of molecular pathways that could directly lead to diabetic cardiomyopathy [14, 16, 20, 22].

In accordance with the above-mentioned results in individuals with type 2 diabetes, we found that empagliflozin, particularly, the empagliflozin-metformin 12-week treatment, acted antioxidatively, the effect of the combination being enhanced to the level where all studied antioxidative parameters (TAS, GPx, SOD) significantly increased with a concomitant decrease in the studied prooxidant parameters (AOPP and isoprostane), compared to the individual drugs or the control group. This concludes in favour of the powerful antioxidant activity of this combination, addressing both sides of the redox spectrum favourably. The anti-inflammatory effect was also the most powerful with the empagliflozin-metformin combination, with a decrease in both CRP and IL-6. In addition to confirming the antioxidative and anti-inflammatory action of the empagliflozin-metformin combination, we also found that the beneficial effects were associated with an improvement in endothelial function and a decrease in arterial stiffness. Therefore, these findings can be proposed as important mechanisms for preventing atherogenesis.

In diabetic patients, regardless of the type, the multifactorial treatment, i.e., treatment of hyperglycaemia as well as regulation of blood lipid levels and hypertension, in addition to smoking cessation and weight loss, leads to significant reduction in major adverse cardiovascular events (MACE) [23, 24]. Therefore, this remains paramount for holistic diabetes treatment. Additionally, antidiabetic drugs with proven cardioprotective action are gaining their place alongside other described multifactorial actions [25]. There is still some debate about whether these drugs should be prescribed as the first- or second-line therapy in individuals with type 2 diabetes. Diabetologists advocate for their second line use, as metformin possesses great antihyperglycaemic activity with proven additional beneficial effects [26]. On the other hand, cardiologists on both sides of the Atlantic believe first-line treatment to be the most desirable [27, 28]. The present study could clarify the answer to this important question. Based on our results, a combination of metformin and SGLT-2 inhibitor could be the most powerful option for glycaemic control and protection of the arterial system through antioxidative and anti-inflammatory actions. The beneficial effects of the empagliflozin-metformin combination observed in our study were obtained in previously drug-naïve individuals for cardioprotective antidiabetic therapy; therefore, the effects could be attributed solely to the studied drug combination. The antioxidative and anti-inflammatory effects of this drug combination not only have antiatherosclerotic benefits but could be extrapolated for systemic effects, attenuating arterial ageing and degenerative processes in several tissues and organs. As described earlier, adults with type 1 diabetes were recruited only because they were oral antidiabetic drug-naïve. Since they had long-standing diabetes, we strongly believe that the results and observed beneficial effects could also be further extrapolated to individuals with type 2 diabetes who would, due to their inclination towards accelerated atherosclerosis even before the diabetes diagnosis, probably benefit the most. This treatment is, in type 2 diabetes, safe and simple, thus allowing for it to be prescribed in different clinical settings. However, despite their high efficiency, the risks associated with the use of SGLT-2 inhibitors in individuals with type 1 diabetes should be also borne in mind [29]. In our study, blood ketones were carefully monitored throughout, with values not reaching the levels of detection. Under nonstudy conditions or in regular clinical practice, the risk of diabetic ketoacidosis exists; therefore, the prescription of SGLT-2 inhibitors in individuals with type 1 diabetes is not particularly advised [29].

A limitation to this study is the relatively small number of enrolled participants and the short duration without any additional follow-up, which did not allow for the determination of MACE. In addition, regression analysis between the risk factors and delta values of FMD was not performed, due to the small number of participants and small number of risk factors per participant. Also, only one dose of empagliflozin, i.e., maximal dose of 25 mg daily, was used, not allowing for comparing the effects of the clinically used empagliflozin doses (10 and 25 mg daily). The study was also not designed to explore the minimal time to effect; thus, we could not determine how fast the beneficial effects of the drugs occurred. Nevertheless, an important strength of this study is that, for the first time, the complex connections between antioxidants, prooxidants, inflammatory parameters, and arterial function parameters were explored.

## 5. Conclusions

In summary, the empagliflozin-metformin combination possesses powerful antioxidative and anti-inflammatory capacity in adults with type 1 diabetes that could, at least partially, underlie the important vascular benefits of this drug combination. These findings add to the already established cardiovascular pleiotropic benefits of these drugs in diabetes.

## Figures and Tables

**Table 1:** Baseline characteristics of the participants at inclusion to the study according to the study groups: control group (CTRL), empagliflozin 25 mg daily group (EMPA), metformin 2000 mg daily group (MET), or empagliflozin-metformin combination group (EMPA-MET COMB).

	CTRL (*n* = 10)	EMPA (*n* = 10)	MET (*n* = 10)	EMPA-MET COMB (*n* = 10)
Average age (years)	43.1 ± 2.1	46.0 ± 2.3	46.4 ± 3.9	43.3 ± 2.6
Type 1 diabetes duration (years)	22.4 ± 3.5	21.8 ± 4.0	23.1 ± 3.9	23.2 ± 4.3
BMI (kg/m^2^)	28.3 ± 0.5	28.9 ± 0.7	28.0 ± 0.3	28.9 ± 0.9
Waist circumference (cm)	96.9 ± 3.9	101.4 ± 2.9	97.8 ± 4.0	99.1 ± 3.9
Baseline HbA1c (%)	7.8 ± 0.2	7.8 ± 0.1	7.9 ± 0.2	7.8 ± 0.2
Smokers	2 (20%)	2 (20%)	2 (20%)	2 (20%)
Arterial hypertension	4 (40%)	3 (30%)	3 (30%)	4 (40%)
Dyslipidaemia	2 (20%)	3 (30%)	2 (20%)	3 (30%)
Diabetic kidney disease	2 (20%)	3 (30%)	2 (20%)	2 (20%)
Diabetic retinopathy	4 (40%)	5 (50%)	4 (40%)	4 (40%)
Diabetic neuropathy	1 (10%)	2 (20%)	1 (10%)	1 (10%)
Previous cardiovascular events	1 (10%)	1 (10%)	1 (0%)	1 (10%)

Values are expressed as mean ± standard error of the mean or number of patients in separate groups. Number of participants with a condition (smoking, arterial hypertension, dyslipidaemia, diabetic kidney disease, diabetic retinopathy, diabetic nephropathy, and previous cardiovascular event) is stated in addition to their relative share in a particular group. BMI: body mass index; HbA1c: glycated haemoglobin.

## Data Availability

The raw data used to support the findings of this study are available from the corresponding author upon request.
